# The Role of Extracellular Matrix Components in Inflammatory Bowel Diseases

**DOI:** 10.3390/jcm10051122

**Published:** 2021-03-08

**Authors:** Alicja Derkacz, Paweł Olczyk, Krystyna Olczyk, Katarzyna Komosinska-Vassev

**Affiliations:** 1 Department of Clinical Chemistry and Laboratory Diagnostics, Faculty of Pharmaceutical Sciences in Sosnowiec, Medical University of Silesia in Katowice, 41-200 Sosnowiec, Poland; derkacz.alicja@gmail.com (A.D.); olczyk@sum.edu.pl (K.O.); 2 Department of Community Pharmacy, Faculty of Pharmaceutical Sciences in Sosnowiec, Medical University of Silesia in Katowice, 41-200 Sosnowiec, Poland; polczyk@sum.edu.pl

**Keywords:** inflammatory bowel disease, ulcerative colitis, Crohn’s disease, extracellular matrix components, hyaluronan

## Abstract

The remodeling of extracellular matrix (ECM) within the intestine tissues, which simultaneously involves an increased degradation of ECM components and excessive intestinal fibrosis, is a defining trait of the progression of inflammatory bowel diseases (IBDs), which include ulcerative colitis (UC) and Crohn’s disease (CD). The increased activity of proteases, especially matrix metalloproteinases (MMPs), leads to excessive degradation of the extracellular matrix and the release of protein and glycoprotein fragments, previously joined with the extracellular matrix, into the circulation. MMPs participate in regulating the functions of the epithelial barrier, the immunological response, and the process of wound healing or intestinal fibrosis. At a later stage of fibrosis during IBD, excessive formation and deposition of the matrix is observed. To assess changes in the extracellular matrix, quantitative measurement of the concentration in the blood of markers dependent on the activity of proteases, involved in the breakdown of extracellular matrix proteins as well as markers indicating the formation of a new ECM, has recently been proposed. This paper describes attempts to use the quantification of ECM components as markers to predict intestinal fibrosis and evaluate the healing process of the gut. The markers which reflect increased ECM degradation, together with the ones which show the process of creating a new matrix during IBD, allow the attainment of important information regarding the changes in the intestinal tissue, epithelial integrity and extracellular matrix remodeling. This paper contains evidence confirming that ECM remodeling is an integral part of directional cell signaling in the progression of IBD, and not only a basis for the ongoing processes.

## 1. Introduction

Inflammatory bowel diseases (IBD), including two main conditions: Crohn’s disease (CD) and ulcerative colitis (UC) are chronic and relapsing inflammatory diseases of the gastrointestinal tract, which have become a global healthcare burden in the 21st century. In CD, the inflammation may occur in any part of the gastrointestinal tract; however, it is usually located in the last region of the ileum and the colon, and the lesions usually stay next to healthy regions of the intestine [1]. In UC, on the other hand, the highest severity of the inflammation and pathological changes is observed in the rectum and decreases from the distal part of the bowel, until an abrupt switch to a healthy tissue. The inflammation in CD may affect the intestinal wall along its whole depth (from mucosa to serosa); in UC, it is usually restricted to the mucosa and submucosa [2]. One of the defining characteristics of inflammatory bowel diseases is the increased remodeling of the extracellular matrix. An excessive and prolonged inflammatory response occurring in the intestinal tissue is a cause of progressive changes to the structure and functioning of the intestinal tissue extracellular matrix [1,2]. 

The extracellular matrix is a highly dynamic structure present in all tissues, which undergoes controlled remodeling. During this process, both quantitative and qualitative changes of its components take place in order to control homeostasis and tissue architecture [3]. Matrix components include: (1) collagen proteins: type I collagen, basement membrane collagens (type IV, VIII and X), type VI microfibrillar collagens, (fibril-associated collagens with interrupted triple helices (FACIT); (2) non-collagen ECM proteins (elastin, fibronectin, laminin, thrombospondin or tenascin; (3) proteoglycans (PG) and glycosaminoglycans (GAG), and (4) growth factors, enzymes, including matrix metalloproteinases. The extracellular matrix is not only a scaffold for cells within the tissues, but also a dynamic component of the tissue, involved in multiple molecular pathways and processes, such as cellular proliferation, migration and adhesion [1,2]. The structure of ECM undergoes constant deposition, degradation or modification. The changes in the extracellular matrix, or so-called ECM remodeling, are caused by specific enzymes responsible for its degradation and rebuilding, such as: matrix metalloproteinases (MMPs), neutrophil elastases (NE) and meprins. MMPs are the main enzymes involved in ECM degradation. Normally, their activity is low, and its increase can be observed during repair or remodeling processes, or during inflammation. The progression of IBD involves a disturbance to the proteolytic–antiproteolytic balance, which leads to increased degradation of ECM components, in which MMPs take part [3]. Apart from increased proteolytic activity, ECM remodeling during IBDs is also related to wound healing and involves the creation of a new ECM, which involves a particularly large increase in the synthesis of fiber-forming collagens (type I, III and V collagens), and FACIT-type collagens (type XII, XVI and XIX). Excessive ECM formation leads to a progressive intestinal fibrosis, which in turn causes intestinal lumen narrowing [1,2,3]. Fibrosis occurs through the activation of fibroblasts and the secretion of ECM components, which bind through the activity of lysyl oxidase (LOX). The increased stiffness of the fibrotic tissue, on the other hand, leads to further fibrogenesis. Progressive tissue damage and IBD-related complications—resulting from unbalanced and deregulated ECM remodeling—cause the disappearance of barriers between the epithelial and the endothelial tissue and contribute to the formation of fistulas, eventually leading to intestinal perforation. The remodeling of extracellular matrix components within the intestines, which involves an increased degradation of ECM components and excessive intestinal fibrosis, is a characteristic feature of the progression of inflammatory bowel diseases. The changes within the extracellular matrix, through various molecular mechanisms, contribute to tissue damage over the course of IBD [4]. 

## 2. The Role of MMPs in Extracellular Matrix Remodeling in the Progression of Inflammatory Bowel Diseases

Metalloproteinases are a group of zinc-dependent endopeptidases, whose main function is remodeling extracellular matrix components. These enzymes are synthesized by fibroblasts and other types of connective tissues cells, as well as by leukocytes, monocytes, macrophages and endothelial cells, and then released into the extracellular space in an inactive form (proMMP). The enzyme is activated by proteolytic cleavage in the propeptide region. The activity of metalloproteinases is precisely regulated at the transcription and translation level, and by endogenous inhibitors, such as α2-macroglobulin and tissue inhibitors of metalloproteinases (TIMPs) [4,5,6,7]. In physiological conditions, MMPs participate in processes such as embryogenesis and angiogenesis, allow the migration of inflammatory response cells to the damaged tissues, release cytokines and their receptors from cell membranes, and regulate the activity of blood platelets. Changes in MMP activity have been observed during many pathological states involving an increased intensity of inflammatory processes [8,9,10,11]. Metalloproteinases are also significant factors during IBD pathogenesis, since they promote inflammation and the degradation of ECM components, causing excessive damage to intestinal tissues. The research conducted so far has pointed to an increased expression of metalloproteases responsible for matrix remodeling in IBD patients [4,5,6]. 

The substrates for metalloproteinases are extracellular matrix proteins, including collagens, fibronectin, laminin, vitronectin or tenascin. The increased MMP activity in IBD patients promotes ECM degradation and causes an increase in the amount of its components released into circulation. In response to proinflammatory cytokines and interactions between cells—or between cells and the extracellular matrix—MMPs are regulated at the transcription level. The most investigated MMPs in IBD are collagenases (MMP-1, MMP-8, MMP-13), gelatinases (MMP-2, MMP-9), stromelysins (MMP-3, MMP-10), matrilysin (MMP-7) and macrophage elastase (MMP-12). The role of individual metalloproteinases in modulating the progression of IBD is summarized in Table 1 [5,6,7,8,9,10,11,12,13].

A group of **collagenases** (MMP-1, MMP-8, MMP-13) plays an important role in IBD progression, and they only occur in inflamed tissues [14]. MMP-1 prevents intestinal fibrosis, which occurs when the synthesis of the extracellular matrix exceeds its degradation. MMP-1cleaves mature collagen fibers, promoting fibrin degradation [5,15]. MMP-8 also has this property; it also participates in neutrophil infiltration into intestinal tissues. Neutrophils release MMP-8 into the inflammatory environment, where it further modulate proinflammatory cytokines and chemokines, enabling the recruitment of further leukocytes [14].

In turn, the proinflammatory role of MMP-13 is due to its ability to activate endostatin and release TNF-α from the membranes, which affects the integrity of the intestinal epithelial barrier [16].

**Gelatinases** (MMP-2, MMP-9) are responsible for most processes throughout IBD progression, including collagen breakdown, similar to collagenases. This action prevents intestinal fibrosis and neutrophil infiltration into the intestinal tissue. MMP-2 participates in remodeling of collagen structures [5]. In IBD pathogenesis, MMP-2 deficits can lead to a deregulation of the intestinal barrier functions and to fibrosis; in case of increased expression, an excess of MMP-2 may induce the formation of fistulas, as seen in Crohn’s disease [14]. MMP-9 plays a similar role to MMP-2 in terms of fistula creation, but MMP-9 may also promote tissue damage by activating neutrophils in regions subject to acute inflammation and by accelerating the proteolysis of matrix proteins partially degraded by other MMPs. MMP-9 participates in the inflammatory response, slows down epithelial repair process, hinders wound healing, increases endothelium permeability and activates cytokines and chemokines, including interleukin IL-1β, IL-8, and TGF-β [7,14].

**Stromelysins** (MMP-3 and MMP-10) play an important role in wound healing, as they cause ECM protein degradation, which allows the endothelial cells, epithelial cells and fibroblasts to detach and reconnect to the ECM in a controlled manner [14].

They also facilitate cell migration through the damaged tissue, which promotes angiogenesis, re-epithelialization and granulation of wounds. Furthermore, MMP-3 causes the release of endostatin from collagen particles; it strongly inhibits the proliferation of endothelial cells, migration and angiogenesis [19]. MMP-7 takes part in endostatin production; furthermore, this matrilysin has an indirect antibacterial effect, as it activates defensins and induces their release from Paneth cells into the intestinal lumen [14,18]. Among other metalloelastases, MMP-12 is also noteworthy, since an increase in its expression has a relation to IBD, as it participates in the immunological response of lamina propria macrophages. MMP-12 is necessary for the migration of macrophage, which degrade various proteins, causing the degradation of intestinal epithelium [14,15].

During inflammation or healing processes within the intestine, an overexpression of a number of MMPs, undetectable in the healthy gut (MMP-1, MMP-8, MMP-9, MMP-10, MMP-12, MMP-13) is observed. It has been shown that increased MMP-1 expression can be seen in the mucous membrane of the colon in IBD patients and it correlates with an exacerbation of the inflammation [17,20]. The intestinal stromal cells, including fibroblasts, myofibroblasts, pericytes, endothelial cells, and smooth muscle cells, have been shown in human colonic myofibroblasts and in IBD patients to secrete MMP-2 during the activation of inflammatory processes and after a stimulation by MMP-1, MMP-3 and MMP-9 [21,22,23]. It has been shown that MMP-3 and MMP-9 are two key enzymes involved in the degradation of intestinal tissue during CD and UC. A significant increase in MMP-3 expression has been noted in the inflamed regions of the colon in IBD patients, compared to the non-inflamed regions [24]. The cleavage of collagen XVIII by MMP-3 leads to increased generation of biologically active endostatin fragments, which are antiangiogenic factors. The significant role of metalloproteases in ECM remodeling has also been confirmed during subsequent investigations, which have shown that the epithelium of the colon in IBD patients produced increased quantities of MMP-1, MMP-3, MMP-7, MMP-9, MMP-10 and MMP-12 [25]. Other studies in the intestinal tissue from IBD patients and mice with dextran sodium sulfate-induced colitis have shown that macrophages are the main source of MMP-8, MMP-9 and MMP-10 during IBD, which suggests a significant role of neutrophils in intestinal tissue infiltration [6,10]. It has been shown that MMP-8 and MMP-9 are stored in neutrophil granules and released into the inflammatory environment, where they modulate the action of pro-inflammatory cytokines and chemokines, which enables the recruitment of further neutrophils to the site of injury. Studies so far have found that serum MMP-9 levels were higher in both UC and CD patients compared to controls and correlated well with the disease activity [26,27,28]. Increased MMP-9 expression and activity promote ECM degradation, which can enhance inflammatory cell infiltration. MMP-10 and MMP-7 matrilysin play a role in intestinal wound healing. The expression of MMP-12 was also increased in IBD patients, which lead to increased degradation of intestinal epithelial cells [13]. It has also been shown that MMP-13 occurred in the inflamed regions of the colon in IBD patients, while it was not present in non-inflamed intestines or during acute diverticulitis, and its expression correlated with the degree of inflammation visible in the histological image of the tissues [29]. Although the main role of metalloproteinases involves the degradation of ECM proteins and basal membrane proteins, these enzymes—including MMP-3, MMP-8, MMP-9 and MMP-7—also participate in inflammatory processes. In addition, the activity and expression of MMPs altered in the course of IBD affects cell adhesion, migration of immune cells, cytokine synthesis and wound healing [6,7,8,9,10,11,12].

Apart from MMPs, the process of extracellular matrix remodeling throughout IBD also involves neutrophil elastases and meprins. Neutrophil elastase is a serine protease, produced mainly by neutrophil granulocytes. Its activity is higher during inflammation, which causes increased elastin degradation and promotes the inflammatory process even more. The third discussed group of enzymes—meprins—are zinc-dependent proteases necessary for the homeostasis of gut microbiome. They are responsible for the breakdown of ECM proteins, such as type IV collagen, laminin and nidogen. On one hand, proper intestinal tissue metabolism requires proper functioning of cells responsible for the synthesis of ECM components; on the other hand, it depends on the activity of specific proteases which degrade them. In a properly functioning tissue, the activity of preteolytic enzymes is subject to complex control processes. MMP expression and the balance between their levels and the levels of tissue metalloproteinases inhibitors is crucial for proper homeostasis of the extracellular matrix in intestinal tissues. Disturbing this balance may lead to intestinal fibrosis. Intestinal fibrosis is a common complication in patients with long-term IBD and interleukin-13 is a potent inducer of tissue fibrosis in IBD patients [29]. Various studies indicate a pathogenic role of MMP-9 in animal models of intestinal inflammation. Of the matrix metalloproteinases, especially MMP-9 has been shown to be consistently increased in different animal models of colitis, such as T cell–mediated colitis, colonic damage induced by dextran sodium sulfate (DSS) treatment, trinitrobenzene sulfonic acid-induced colitis, and human colitis as well [24,30,31,32]. These data confirm that MMP-9 is an important marker of inflammation in IBD [30,31]. It has been also found that MMP-9 knockout mice are resistant to the development of experimental colitis [28]. Increased expression of MMP-1, -3, -7, -9, -10 and -12 has been demonstrated in human colon epithelium in the intestinal tissue from IBD patients [10,14,18]. Mucosal biopsies of UC patients showed the presence of vascular endothelial cells and infiltrating leukocytes as the main sources of MMP-7 and -13 [32]

## 3. The Role of MMP in the Immunological Response

The processes catalyzed by the enzymes which participate in rebuilding the ECM are irreversible, and the activity of enzymes has to be regulated at the level of transcription, translation, secretion, and activation; it is limited by metalloprotease inhibitors within the tissues. This introduces a higher level of control, mainly in the regulation of the immune system. The proteolysis of ECM and basal membrane proteins, which involves ECM proteases, makes it easier for immune cells to migrate up to the mucous membrane of the intestine [24,25]. The proteolysis-related change in the structure of ECM components also affects their functioning. The ECM fragments released via degradation processes influence the expansion of the cells which bind them—macrophages and lymphocytes—making it easier to remove them from circulation and migrate to the inflamed regions. Prolonged deposition of these activated cells increases the intensity of the inflammation. Due to the great number of ligands capable of interacting with ECM particles, it is believed that changes to their structure may cause disturbance to the microenvironment and the dynamics of immune response, affecting proper immune processes and leading to the development of IBD. Damage to ECM structure allows the interaction of microbial antigens and the immune system, exacerbating the chronic inflammation related to IBD. Furthermore, some ECM proteins act as inflammatory factors. It has been proven that increased gelatinase B activity, through its involvement in ECM proteolysis and the promotion of immunological signaling, supports IBD development. It works due to the proinflammatory cycle in the intestines. The activity of gelatinase B induces the formation of a product of collagen breakdown: proline–glycine–proline (PGP). It participates in neutrophil chemotaxis and induces the expression of gelatinase B [28]. The increased gelatinase B concentration in the extracellular space leads to the exacerbation of the inflammation in the intestine [33]. Another vital mechanism suggesting a regulatory function of ECM integrity in IBD pathogenesis is the signaling which occurs through hyaluronan (HA), which controls the recruitment of immune system cells, release proinflammatory cytokines and participates in hemostasis.

Research indicates that single nucleotide polymorphism (SNP) within the promoters of genes encoding metalloproteinases influences the expression of these enzymes and therefore the development of IBD [34,35,36,37,38]. Phenotypic analysis of the SNPs revealed associations with various measures of disease severity such as: age at first onset, location of pathological changes, extra intestinal manifestations and the requirement for bowel resection [34,37]. Studies have shown several SNPs in MMP-10 gene significantly associated with UC, for example Rs4431992 polymorphism was associated with extra-intestinal manifestations and Rs12290253 was associated with the female gender, age at first diagnosis above 40 years and pancolitis [34]. Moreover research revealed SNPs in MMP-8 gene which were also significantly associated with UC. Other studies suggest that the MMP-3 SNP can also be associated with increased risk of stenosing behavior in CD [35]. Although further studies are needed to determine the robustness of these observations.

Researchers also bring attention to the MMP inhibitors that show therapeutic potential in the treatment of IBD. The tested inhibitors are expected to reduce the expression of MMPs and therefore positively affect the course of the disease [37,39,40]. For example, administration of mesalamine may result in reduced MMP-2 expression and inflammation in the course of UC [39]. Moreover, treatment of CD patients with immunosuppressives such as methotrexate, corticosteroids or anti-TNF therapeutics resulted in a decrease in epithelial MMP-7 and stromal MMP-9 and -26 which also led to improvement of patients [40]. The therapeutic properties of MMP inhibitors prove that MMPs greatly determine the onset and course of IBD.

## 4. The Role of Hyaluronan in the Progression of Inflammatory Bowel Diseases

**Figure 1:**
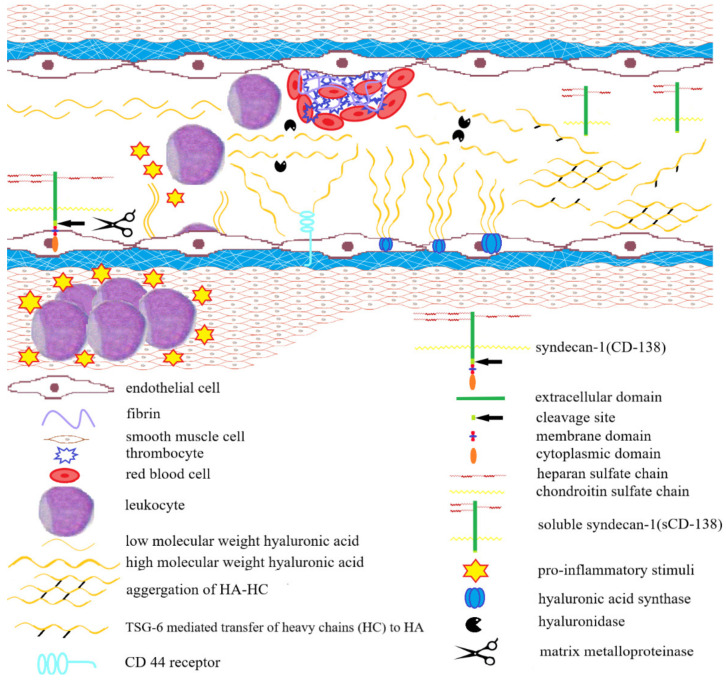
The regulatory role of hyaluronan in the progression of inflammatory bowel diseases. While induced by inflammatory stimuli, synthesis of hyaluronan (HA), an abundant component of the extracellular matrix, increases. On the other hand, in chronic inflammatory conditions such as inflammatory bowel diseases (IBD), high molecular weight HA (HMW-HA) is degraded into fragments that are capable of induces signaling inflammatory responses via specific receptors, induces angiogenesis and inhibits fibroblasts differentiation. Moreover, throughout IBD, HA forms complexes with trypsin inter-α inhibitor (HA–HC matrix), which are highly adhesive towards leukocytes and exacerbate the inflammation.

The regulatory role in maintaining ECM integrity during IBDs is played by molecular mechanisms based on signaling pathways which involve hyaluronan (HA). Hyaluronan is an unbranched, unsulfated glycosaminoglycan, whose polymerization level reflects the integrity of the HA matrix; the presence of fragmented HA parts with a low molecular weight, on the other hand, reflects its degradation. HA participates in wound healing, proliferation and migration of cells and modulating the inflammatory process. This unsulfated GAG which does not form covalent bonds with proteins, can also bind water in its polysaccharide net, providing proper hydration to the matrix, which allows cell migration and substance diffusion. Hyaluronan actively participates in many diseases, including IBD, and its level increases in the inflamed regions. During excessive inflammation, these polymers are cleaved to fragments of lower molecular weight that take on signaling roles [41]. The regulatory role of HA in chronic inflammatory conditions associated with IBD, has been presented in Figure 1.

As an ECM component, hyaluronan has shown minimal adhesive properties towards leukocytes in proper conditions; throughout IBD, not only is there an increase in its synthesis, but it also forms complexes with trypsin inter-α inhibitor, which are highly adhesive towards leukocytes and exacerbate the inflammation.

The deposition of hyaluronan fragments in the intestine is related to the inflammatory state during IBD [41,42,43]. Certain cell types relevant to the pathobiology of IBD, such as human smooth muscle and intestinal vascular endothelial cells, and porcine intestinal epithelial cells, have been observed to positively influence monocyte adhesion by producing HA chains in response to inflammatory stimuli [44,45]. Therefore, the proinflammatory signals induced by e.g., TNF-α lead to increased synthesis of hyaluronan, forming an HA matrix with strong adhesive properties [46,47]. The biosynthesis of hyaluronan matrix is made possible by tumor necrosis factor-stimulated gene 6 (TSG-6), which is a protein weighing 35 kDa. It catalyzes the movement of heavy chains (HC) from a serum proteoglycan complex, inter-alpha-inhibitor, to a hyaluronan particle, which leads to the creation of an HA–HC matrix [48,49]. TSG-6 expression is induced in the presence of inflammation [4]. Hyaluronan—in the form of an HA–HC matrix, as opposed to an HA matrix—which shows adhesive properties towards blood platelets and leukocytes [49,50,51].

Thus, the deposition of hyaluronan fragments and HA–HC matrix with strong adhesive properties induces the infiltration of leukocytes towards the intestinal lumen, which leads to the activation of an inflammatory response [42,43]. On the other hand, the accumulation of HA fragments with a low molecular mass also influences wound healing by inducing fibroblast proliferation and myoblast differentiation, which in turn influences the fibrosis processes during IBD. The accumulation of HA fragments confirms the active role of the ECM in commonly known pathogenic pathways responsible for initiating intestinal pathology and IBD progression [52,53].

Furthermore, studies on mice and rats have shown that HA synthesis precedes the infiltration of inflammatory cells and therefore promotes inflammation. For this reason, controlling HA levels on the surface of cells may play a regulatory role during IBD [47]. The chronic inflammation typical of IBD results in a significant deposition of HA in the affected tissues, which both precedes and promotes the infiltration of immune cells, tissue destruction and blood coagulation. Perhaps the balance between HA synthesis and degradation and the interactions between this matrix component and binding proteins are the controlling factors in the recruitment of inflammatory cells by the HA–HC matrix [47,48,49].

The role of hyaluronan in IBD pathophysiology is also related to the fact that it possesses the ability to regulate the function of intestinal epithelium, whose integrity is vital for preventing the formation of blood clots [52,54,55,56,57]. In IBD patients, as opposed to healthy people, microvascular closure in the mucous membrane is observed [58]. The characteristic features of IBD are: fluctuations in the platelet reactivity, increased coagulation and an increased risk of arterial and venous thrombosis [58,59]. The risk of blood clots becomes apparent during the remission phase and increases with the activity of IBD. Inflammation mediators TNF-α and IL-6 have the ability to initiate coagulation and activate HA synthesis [60,61]. Fibrinogen, a protein which participate in the final part of the clotting process and is converted to fibrin, also has the ability to bind HA [62,63,64]. As a result, it leads to the formation of hydrated matrices and an increased influx of inflammatory cells into the clot [65,66].

It has also been shown that thrombin, which participates in the coagulation and is necessary for converting fibrinogen to fibrin, can also interact with HA–HC fragments [67]. Furthermore, the increased amount of HA in IBD patients leads to inhibited synthesis of antitrombin—a protein belonging to serine protease inhibitors, whose function involves inhibiting coagulation factors such as thrombin, coagulation factors IXa, XIa, XIIa, or kallikrein [64,65,66]. On the other hand, in vitro studies on blood platelet functions have proven that purified hyaluronan has the ability to inhibit adhesion and aggregation of thrombocytes, which prevents blood clot formation [54,65,66,67]. Due to its engagement in multiple molecular mechanisms, hyaluronan may promote blood coagulation and the recruitment of leukocytes during IBD progression [43].

In summary, hyaluronan participates in inducing and maintaining the inflammation, in the coagulation process, and in modulating the remission and activity of the disease. Inflammation and coagulation are two interconnected processes which reinforce and support each other, finally leading to the inhibition of damage progression. Molecules which mediate inflammation and clotting, such as HA, may contribute to both weakening and aggravating disease, depending on the structure. The HA–HC also supports the recruitment of inflammatory cells. A prolonged inflammation leads to impaired epithelial function, increased thrombus formation, tissue damage and an increased disease activity [43,60,61,62,63].

## 5. Other Extracellular Matrix Components Involved in the Inflammatory Process during IBD

### 5.1. Laminin (LN)

In order to detect changes in the extracellular matrix in the course of IBD, attention was also paid to the potential diagnostic and/or prognostic utility of determining other components of the extracellular matrix in the blood. One of the new diagnostic markers is the concentration of **laminin (LN)** determined in the serum of IBD patients [68,69,70,71]. Laminin is synthesized by epithelial cells and stromal cells of the intestines. This protein allows maintenance of the structure and function of the basement membrane [71]. It was found that the concentration of LN in the blood serum of patients with CD is higher than in the control group and is related to the disease activity. During inflammatory bowel diseases, one can observe an overexpression of laminin Lm-α1 and Lm-α5 cells in colon tissue. In patients with Crohn’s disease, increased expression of the Lm-α1 chain was found in the crypts of the small intestine, both in inflammatory and non-inflamed sites, while increased expression of the Lm-α3 chain appeared only in the crypts of the small intestine that were inflamed [68,69,70].

### 5.2. Heparan Sulfate Proteoglycans (HSPG)

One of the most investigated ECM components of the intestinal tissues are also heparan sulfate proteoglycans (HSPG). It has been shown that HSPGs promote intestinal regeneration, which suggests that they play a role in the homeostasis of intestinal stem cells (ISCs). The HSPGs, whose presence has been noticed on the basal surface of epithelial cells and which play a vital role in maintaining proper functions of the intestinal barrier, include syndecans [72,73,74]. The biological functions of syndecans depend on the presence of glycosaminglycans and include the activation and binding of growth factors, as well as participation in cell adhesion. Syndecans contain from three to five heparan sulfate chains and chondroitin sulfate chains, which allow them to interact with many different ligands, including the fibroblast growth factor, vascular endothelium growth factor, fibronectin and antitrombin-1 [75]. The cytoplasmatic domain of syndecan enables the creation of connections between the cell and the extracellular matrix. Syndecan-1 (CD138) acts as a co-receptor of multiple extracellular ligands, including proinflammatory cytokines and growth factors. Due to these properties, it plays a vital role in many metabolic processes, such as: remodeling the matrix, repairing tissues, regulating the immune system and the progression of inflammatory processes. Within the intestines, syndecan-1 is also responsible for maintaining epithelial integrity and permeability, and protecting enterocytes during their interactions with bacteria. An extracellular fragment of syndecan-1 (sCD138) is released from the surface of cells in a proteolytic, continuous manner, in a process known as ectodomain shedding. The process is activated in response to inflammation, pathogen infection, and wound healing. The presence of the extracellular domain of syndecan-1 in blood might therefore be a precious marker of the activity of inflammatory processes. Pioneering studies have shown [76] that sCD138 concentration in serum during Crohn’s disease is higher than in the healthy population and correlated with the intensiveness of inflammation, which makes it a reliable marker of disease activity. Increased sCD138 concentrations have also been noticed in UC patients, accompanied by an increased level of tumor necrosis factor alpha (TNF-α) and neutrophil amount. It is suggested that releasing the extracellular domain of syndecan-1 from the intestinal epithelial cells may reduce the intensity of inflammatory bowel diseases and the transmigration of neutrophils by deactivating key inflammatory mediators and reducing the expression of proinflammatory cytokines. The protective effect of the syndecan-1 extracellular domain circulating in blood has been confirmed during research on mice lacking sCD138; when subjected to an experimental colitis, they have exhibited significantly increased mortality, impaired mucous membrane regeneration and extended inflammatory cell recruitment. Furthermore, treating animals with a functional analog of the sCD138 ectodomain significantly affected the symptoms of the inflammation [72,73,74,75,76,77].

### 5.3. Fibronectin (FN)

Other proposed biomarkers of intestinal fibrosis include the level of extracellular matrix proteins in serum, including fibronectin. Fibronectin (FN) is an extracellular glycoprotein which binds with various matrix components [68]. Throughout IBD progression, this glycoprotein participates in interactions between the cell and the matrix. It is responsible for fibroblast migration and proliferation through regulating the bioavailability of TGFβ. FN also binds with TNFα, influencing chemotaxis and MMP-9 expression in monocytes. The concentration of fibronectin in the blood was assessed in patients with inflammatory bowel disease. It has been shown that fibronectin concentration in the blood of CD and UC patients differs from those of the healthy population. In CD patients, this marker was significantly lower during active inflammation than in remission [78,79,80,81].

### 5.4. Sulfated GAGs

Characteristic for inflammatory bowel diseases, the process of intensified and abnormal remodeling of ECM components is associated with an excessive and chronic inflammatory response in the intestinal tissue. In turn, the degree and depth of intestinal lesions can be assessed by quantifying the concentration of blood markers that have been released as a result of increased protease activity [82,83] Keratan and dermatan sulfates (KS and DS) belong to the group of sulfated GAGs of the intestinal epithelium. Their amount, released due to excessive ECM degradation during IBD, may be assessed in body fluids and serve as a biomarker for evaluating ECM function with regards to regulating epithelium permeability. Sulfated GAGs participate in the regulation of inflammatory processes and are involved in regulating the permeability of the intestinal epithelium. A significant loss of sulfated GAGs has been confirmed in the subepithelial basal plate of the tissue samples obtained from IBD (both CD and UC) patients. Another possible pathogenetic mechanism, which affects ECM remodeling observed in IBD patients may be the disturbance of GAG structure and function in the connective tissue, related to the ongoing inflammation [83].

## 6. Extracellular Matrix Components as Biomarkers in IBD Diagnosis

So far, there are no known markers which would allow diagnosis and differential diagnosis between UC and CD and possession of the required sensitivity and specificity. Extending the research on ECM may help to identify a component of the extracellular matrix that could supplement or even replace the currently used invasive, time-consuming and expensive colonoscopy and histopathological techniques. Research has shown that fecal MMP-9 concentration reflects the activity of bowel inflammation and mucosal healing in UC [84]. The research by Farkas et al. has shown a correlation between MMP-9 and the clinical symptoms of inflammation and its activity during ulcerative colitis [85]. An overexpression of MMP-2 in the mucous membrane of the colon in UC patients has also been observed [86]. MMP-9 is connected to disease activity in both UC and CD, although higher MMP-9 levels have been observed for UC. Metalloproteinases have therefore been considered potential biomarkers of disease activity during IBD [87].

Other research involved evaluating the potential diagnostic value of measuring TIMP levels, in tissue samples obtained from intestinal tissues, as markers of inflammation severity during IBD [88,89,90,91]. It is commonly known that UC involves increased concentrations of MMP-1 and TIMP-1 and that their expression in colon walls is correlated with the severity of the disease, while the concentrations of these proteins in serum correlated with their expression in the mucous membrane [78]. MMP and TIMP levels in CD patients have been measured before and after immunosuppressive treatment; it has been observed that the histological result was positively correlated with neutrophil MMP-9 and MMP-26, and macrophage TIMP-1 [88]. Furthermore, gelatinase B activity in the intestinal tissues in IBD patients has shown correlation with the clinical improvement and healing of the intestinal mucous membrane [89,90,91]. In many investigations, gelatinase B has been recently considered a serological, urinal and fecal IBD biomarker, suitable as a tool for diagnosing and monitoring IBD. It has been shown that its levels in the body fluids of patients are positively correlated with other known markers of IBD activity and are influenced by immunosuppressive treatment [92,93].

Raised considerable interest can be observed in relation to glycosaminoglycan/proteoglycan-based diagnostic tools. The most important extracellular matrix component evaluated as a potential IBD biomarker is hyaluronan. This glycan, along with the enzymes and binding proteins responsible for its synthesis and degradation, may directly modulate the progression of the disease by controlling the recruitment of immune cells and releasing proinflammatory cytokines. Hyaluronan particles may therefore modulate both the progression and the remission of the disease. HA particles or the products of its degradation, which stimulate the expression of genes regulating the inflammatory processes, may be a marker of disease progression. Research has shown that the presence of HA–HC complexes may exacerbate the disease. Further research—aimed at evaluating disturbed interactions between immunological cells and HA–HC, direct blocking of the HA–HC matrix synthesis and a selective degradation of HA–HC in experimental models of colitis—are necessary for isolating the molecular paths which allow this polymer to regulate the inflammation and may lead to designating HA–HC a marker of inflammation in IBD [46,47,53,54].

## 7. Conclusions

Inflammatory bowel disease leads to the degradation and fibrosis of tissues. Disturbed ECM remodeling during IBD is closely related to an increased activity of MMPs. This leads to excessive degradation of extracellular matrix components, the release of proteins and glycoproteins that build the extracellular matrix into the local tissue and the circulation. Additionally, ECM remodeling is a key mechanism that contributes to the development of IBD and is associated with the accumulation of HA fragments that enable wound healing and contribute to intestinal fibrosis. Any change involving the ECM affects cellular processes, and since ECM also acts as a reservoir for signaling molecules, such a change also induces signaling pathways, generating major switches in the regulation of immune system and other physiological processes. Further research into the molecular mechanisms underlying the remodeling and degradation of ECM would create the basis for the discovery of a component of ECM that would be useful both for the recognition of CD and UC, and for differential diagnosis.

## Figures and Tables

**Table 1:** The role of matrix metalloproteases in inflammatory bowel diseases.

MMP	Group	Common Name	Role in IBD
MMP-1	collagenases	collagenase-1	• prevents intestinal fibrosis
MMP-8		collagenase-2	• neutrophil infiltration into the intestinal tissue
MMP-13		collagenase-3	• activates TNF-α and endostatin [5,6,7,14,15,16]
MMP-2	gelatinases	gelatinase A	• prevents intestinal fibrosis • produces antiangiogenic factors • supporting epithelial tissue [17]
MMP-9		gelatinase B	• neutrophil infiltration into the intestinal tissue, • chemokine expression • produces antiangiogenic factors • prevents intestinal fibrosis [6,7,8,9,14]
MMP-3	stromelysins	stromelysin-1	• activating endostatin [7,18]
MMP-10		stromelysin-2	• involved in wound healing [10]
MMP-7	matrilysins	matrilysin-1	• activating A-defensins • chemokine expression • endostatin production [7,11,12,18,19]
MMP-12	other metalloproteases	macrophage metalloelastase	• degrades intestinal epithelium [13,14,15]
